# Reliable resting-state EEG connectivity measures reveal brain network destabilization and cognitive decoupling following prolonged extreme working conditions

**DOI:** 10.1007/s11571-026-10443-1

**Published:** 2026-05-21

**Authors:** Samet Çelik, Serap Aydın

**Affiliations:** 1 Department of Psychology, Bartın University, Bartın, Turkey; 2 Department of Biophysics, Faculty of Medicine, Hacettepe University, Sıhhiye, Ankara, Turkey

**Keywords:** Resting state EEG, Graph theory, Functional brain connectivity, Miners, Neurocognitive health, Reliable connectivity

## Abstract

This study aimed to characterize alterations in functional brain network organization in miners with approximately ten years of occupational exposure to extreme working conditions and shift work, using connectivity metrics derived from resting-state EEG recorded during both eyes-open (EO) and eyes-closed (EC) conditions. Directed Transfer Function (DTF), the imaginary part of Coherence, and the weighted Phase Lag Index were computed from non-overlapping 6-s epochs following two preprocessing pipelines: Independent Component Analysis and Artifact Subspace Reconstruction (ASR). Analyses were conducted for both miners (19 men, mean age 36.52 ± 5.08 years) and matched controls (19 men, mean age 35.42 ± 5.04 years). DTF demonstrated consistently excellent reliability (Intraclass Correlation Coefficients ≥ 0.75) and revealed significant group differences across all frequency bands when combined with ASR, as determined by linear mixed-effects models with false discovery rate correction (pc < 0.05), underscoring its high reproducibility. Specifically, miners exhibited reduced network segregation and integration, reflected by decreases in modularity (Q), global efficiency (GE), local efficiency (LE), clustering coefficient (CC), and transitivity (T) in the delta and theta bands, as well as reduced network resilience (R) at higher frequencies in the EC condition. Within the miner group, higher GE was associated with poorer executive function and slower processing speed, as measured by Trail Making Test subcomponents (0.51 ≤ r ≤ 0.71; 0.0008 ≤ pc ≤ 0.029). In addition, lower-frequency network metrics (CC, LE, T, and R) showed significant negative correlations with verbal recall performance (− 0.70 ≤ r ≤ − 0.54; 0.0009 ≤ pc ≤ 0.039). Collectively, these findings indicate that chronic occupational exposure disrupts the stability and large-scale organization of functional brain networks, resulting in reduced network efficiency and a decoupling between neural connectivity and cognitive performance. From a methodological perspective, the combination of DTF and ASR emerged as the most reliable approach for resting-state EEG connectivity analysis.

## Introduction

Over the past two decades, the effects of environmental factors on human health have become an increasingly important focus of scientific research (Berman et al. 2019). In this context, environmental neuroscience has emerged as an interdisciplinary subfield that examines how the environment affects individuals, particularly at the level of the central nervous system (Berman & Bratman, 2024). The work environment, a significant component of the concept of the environment, represents a specific context to which individuals are exposed for extended periods and in an intense manner. Current findings indicate that various physical, chemical, and psychosocial agents encountered in the work environment can significantly influence mood, behavioral patterns, and cognitive processes through the nervous system. In this regard, underground mining sites stand out from other work environments due to the diversity of risk factors they present, offering a unique opportunity to understand how the environment affects the human neural system (Zhang et al. 2024). These agents found in mining environments are known to cause numerous health problems in workers, including sensory loss, various types of cancer, and respiratory system diseases, particularly lung diseases (Lu et al. 2021; Parra-Cortés et al. 2025). However, a recent retrospective cohort study has revealed that miners are not only at risk for physical illnesses; these risk factors, to which they are exposed at an early age, also constitute significant risk factors for various neurological disorders later in life, primarily Alzheimer's disease and motor neuron diseases (Zheng et al. 2020). Given that neurodegenerative diseases typically follow a progressive course, it is necessary to investigate whether these disorders exert measurable effects on the neural system during early stages, prior to the emergence of clinical symptoms. Accordingly, an increasing number of studies in recent years have aimed to determine whether exposure to risk factors among miners affects neural functions even at early stages.

Coal mining involves harsh working conditions, including high physical and mental demands, shift work, elevated temperature and humidity, neurotoxic exposure, and low illumination, all of which can significantly affect miners’ cognitive functioning. Accumulating evidence indicates that these environmental stressors contribute to cognitive decline through increased stress, fatigue, and attentional disturbances. In a comprehensive meta-analysis, Zhang et al. (2024) demonstrated that adverse mining environments are associated with heightened stress and fatigue levels, ultimately leading to neurocognitive impairments (Zhang et al. 2024). Consistent with these findings, Chai et al. (2024) reported that low illumination negatively affects miners’ psychological states and cognitive performance (Chai et al. 2024), while Li et al. (2022) confirmed stress-induced cognitive impairments using a mining-like environment in an animal model (Li et al. 2022).

Neuroimaging studies have further elucidated the neural correlates of these cognitive disturbances. Using functional near-infrared spectroscopy (fNIRS), Tian et al. (2022a) showed that shift work is associated with reduced prefrontal oxyhemoglobin (HbO) concentrations, particularly in the dorsolateral prefrontal cortex (DLPFC) and frontopolar regions, reflecting impaired executive control and decreased alertness (Tian et al. 2022a, 2022b). Extending these findings, Tian et al. (2024b) demonstrated that high temperature–humidity interactions significantly suppress functional connectivity between bilateral DLPFC regions during an alertness task, suggesting disrupted prefrontal network integration and reduced cognitive flexibility and attention under environmental stress (Tian et al. 2022b).

Electroencephalography (EEG) studies have provided complementary evidence of mining-related neurocognitive alterations. Çelik et al. (2024) reported a reduction in event-related alpha-band power during a visual working memory task, which was negatively correlated with the duration of underground work, indicating cumulative cognitive impairment. Similarly, Xu et al. (2023) employed EEG-based relative power spectral topography combined with convolutional neural networks to detect miner fatigue, observing increased delta and theta band activity associated with fatigue states. Additional EEG studies have examined distraction (Kuang et al. 2025) and alterations in mental state using multi-domain data integration approaches (Pan et al. 2025), further supporting the presence of mining-related neural dysfunctions.

Despite these advances, existing neuroimaging approaches remain limited in their ability to characterize large-scale brain communication. fNIRS studies provide valuable insights into localized hemodynamic responses but are restricted to superficial cortical regions and offer limited temporal resolution. Likewise, EEG-based power spectral density and band-ratio analyses primarily quantify local oscillatory activity at individual electrodes or frequency bands, without capturing interactions across distributed neural systems. Consequently, the large-scale network organization underlying miners’ cognitive vulnerabilities remains insufficiently understood.

Graph-theoretical analysis offers a robust framework to address these limitations by enabling the examination of whole-brain functional network topology, including key properties such as segregation, integration, and modularity (Bullmore & Sporns 2012). Although scalp EEG has restricted spatial resolution such that graph nodes represent electrodes rather than specific cortical regions, sensor-level analyses nonetheless allow the characterization of large-scale network properties across frequency bands (Rubinov & Sporns 2010). When combined with robust methodological strategies, such as minimum spanning tree construction and proportional thresholding, graph-based EEG analyses can yield reliable and comparable network measures across individuals, permitting valid inferences about functional network organization without overinterpreting spatial localization.

Previous findings suggest that environmental stressors may disrupt functional brain networks by reducing segregation, impairing integration, and altering modular organization, thereby compromising efficient information processing. While evidence from fNIRS studies (Tian et al. 2022a; 2022b) and EEG power-based analyses (Çelik et al. 2024; Xu et al. 2023) supports this notion, whole-cortex connectivity dynamics in miners have not yet been systematically investigated using graph-theoretical approaches.

In the present study, we therefore examined resting-state sensor-level EEG connectivity in coal mine workers and healthy controls using multiple connectivity measures with different sensitivities to volume conduction effects (Directed Transfer Function, imaginary Part of Coherence and weighted Phase Lag Index). Rather than relying on a single estimator, we adopted a comparative framework to determine which connectivity approach most accurately captures group-related differences in large-scale network organization. In addition, given that connectivity estimates are critically influenced by preprocessing choices, we systematically evaluated the impact of two distinct noise-reduction strategies on connectivity outcomes and derived graph-theoretical metrics. This design allowed us to assess not only neurophysiological differences between groups but also the methodological robustness of connectivity findings across preprocessing pipelines. Graph-theoretical metrics, including modularity, global efficiency, local efficiency, clustering coefficient, transitivity, and assortativity, were derived from eyes-open (EO) and eyes-closed (EC) resting-state EEG data across full-band (0.5–60 Hz) and canonical frequency sub-bands.

The primary aim of this study was to determine how prolonged exposure to adverse working conditions in coal miners alters large-scale brain network organization, with particular emphasis on changes in network segregation, integration, and modularity, and to identify the EEG frequency bands most sensitive to these alterations. By integrating a comparative methodological evaluation with an application to an occupationally at-risk population, the study offers both methodological and domain-specific contributions to the literature on resting-state EEG connectivity.

## Methods

The analysis of resting-state surface EEG data, collected in a single session from each participant under EO and EC states, was conducted separately for each condition. The initial 10 s of each condition’s recording were discarded, and the subsequent 4-min segment was windowed and divided into non-overlapping 6-s segments for filtering. We determined this epoch length based on literature findings: In the most recent functional connectivity analyses, to estimate graph theoretic connectivity indices from simulated resting state 128-channel EEG data, mean squared COH, iCOH and wPLI were used for epoch lengths of 2 s, 4 s, 6 s, and 8 s and the number of epochs was varied to be 20, 40, max. As a result of detailed comparisons, it was reported that the most suitable connectivity estimates were obtained for 40 epochs of 6 s length (Miljevic et al. 2025). In previous studies calculating connectivity indices from eyes-closed resting-state 256-channel EEG data collected from the same participants 2 years apart, it was reported that stable results were obtained when the wPLI approach was applied to four 12-s epochs rather than to 4-s epochs (Hardmeier et al. 2014). In another previous study, 128-channel eyes-closed resting-state EEG data were recorded from the same participants one week apart. Connectivity indices were obtained from fixed-length 2-s epochs using the COH and wPLI approaches, and prediction stability was examined in each frequency band. The analyses found that the COH approach produced more stable results in the delta and theta bands, while the wPLI approach produced more reliable results at higher frequencies such as beta. Intra-Class Correlation (ICC) reliability was lower in the gamma band (Kuntzelman and Miskovic 2017). Based on these findings, we adopted an epoch length of 6 s, which provides a balance between capturing sufficient data points for reliable spectral and phase-based estimates. This duration is long enough to ensure stable connectivity and graph indices, but short enough to limit contamination by slow drifts, vigilance fluctuations, or artifacts that are more likely to affect longer epochs. Thus, the 6-s window represents an empirically supported compromise, consistent with previous methodological recommendations for resting-state sensor-level connectivity studies. After segmenting the data into epochs, a notch filter at 50 Hz was applied to suppress powerline interference, followed by a fourth-order Butterworth band-stop filter (60–100 Hz) to attenuate high-frequency EMG activity. And a band-pass filter (FIR filter) was applied sequentially. The band-pass filter's frequency range was adjusted to the targeted EEG sub-band. Other filter parameters were standardized across all frequency bands (pass-band ripple: 0.057501127785, stop-band attenuation: 0.0001, density factor: 20).

In the second step, two distinct approaches were employed to remove noise. In the first approach, the Artifact Subspace Reconstruction (ASR) algorithm suggested in reference (Mullen et al. 2015) was applied, while in the second, Independent Component Analysis (ICA) suggested in reference (Castellanos and Makarov 2006) was used as described in Sect. 2.3. For both approaches, the filtered and artifact-cleaned EEG segments were subjected to connectivity methods (DTF, iCOH, wPLI), which are introduced in the subsequent subsections. The DTF method was implemented using the eConnectome toolbox (Huang et al. 2021), while EEG segment modeling was performed with the *AR-Fit toolbox* (Schneider and Neumaier, 2001). Connectivity indices were calculated from the connectivity values computed for each 2-s segment of the 20-channel surface EEG recording using the Brain Connectivity Toolbox (Rubinov and Sporns 2010. Each method’s implementation steps are detailed and introduced in the following subsections. All computational steps were performed using MATLAB 2025Ra. First, to identify which connectivity estimation method yielded the most stable estimates across repeated epochs, we calculated Intraclass Correlation Coefficients (ICCs) for each frequency-band-specific connectivity index separately for the EO and EC states across 40 non-overlapping 6-s epochs for each participant. After determining the method that provided the most stable indices, we subsequently examined group-level statistical differences in the connectivity indices derived from that method. ICCs were computed using a MATLAB implementation of the Shrout and Fleiss framework for assessing rater and measurement reliability (Shrout & Fleiss 1979. We employed the function provided by Brownhil, in which rows represent raters or repeated measurements and columns represent the individual targets or cases being evaluated (Brownhill 2025). Following the Shrout–Fleiss schema, the ICC model was selected as ICC(3,k)**,** appropriate when each target is measured by the same fixed set of raters and the reliability of the mean rating across raters is of interest. The resulting ICC values were interpreted according to widely adopted thresholds, defining reliability as *poor* for ICC < 0.40, *fair* for 0.40–0.60, *good* for 0.60–0.75, and *excellent* for ICC ≥ 0.75 (Jin et al. 2011; Hardmeier et al. 2014; Kuntzelman and Miskovic 2017).

Statistical differences between the two groups were analyzed using Linear Mixed-Effects models (LMEs) in MATLAB, with a significance criterion of p < 0.05. Regarding ICC values originating from the integration of three methods (DTF, iCOH, wPLI) with ASR and ICA, LMEs were determined for the specified integration, producing estimates with good or excellent reliability (ICC ≥ 0.60). In detail, separate LMEs were constructed for each band-specific connectivity index as described in Sect. 2.5. Finally, the most meaningful estimations were visualized in figures.

*EEG data acquisition and ethical approval*

EEG was recorded from twenty different locations (Fp1, Fp2, F3, F4, Fz, F7, F8, C3, C4, Cz, T7, T8, P3, P4, Pz, P7, P8, O1, O2, and Oz) and electrode placements based on the international 10–20 system. The EEG was amplified by Enobio32 (NeuroElectrics, Spain) with a bandpass of 0 to 125 Hz and digitized online at 500 Hz, with the right earlobe as the reference electrode. The impedance was kept below 10 kΩ during all recordings.

Participants sat in a dimly lit room during EEG recordings. They were seated in a comfortable armchair during the rsEEG recording and instructed to remain awake, with no movement, to avoid focusing on external stimuli, and to avoid cognitive tasks such as planning during recording. Once the participants understood the instructions, a 6-min eyes-open rsEEG recording was first taken. Then, a 6-min eyes-closed rsEEG recording was taken. All procedures conducted in studies involving human participants adhered to the ethical standards set forth by the institutional and/or national research committee, as well as the 1964 Helsinki Declaration and its subsequent amendments or equivalent ethical guidelines. The study protocol received approval from the Istanbul Medipol University Non-Interventional Clinical Research Ethics Committee (Approval No: E-10840098–772-02–897). Prior to data collection, researchers provided participants with a comprehensive explanation of all experimental procedures. The informed consent form, signed by all participants, also stated that the recorded EEG data would be analyzed using computer-based methods for scientific research. Informed consent was obtained from all participants, and the aforementioned ethics committee approved the study.

### Participants

19 mineworkers working underground in a single mine site and 19 control participants matched with these workers in terms of age, working duration, and education level were included in this study. Since working underground is legally prohibited for women in Türkiye, the entire study sample consists of male participants. Within the scope of the study, underground mineworkers were first selected according to predetermined inclusion and exclusion criteria; then, a control group matched for demographic characteristics was selected. Previous studies were used to determine the criteria (Çelik et al. 2025; Çelik, 2025).

Inclusion criteria for mine workers: (I) Having at least 5 years of underground mining experience, (II) Having a minimum primary school degree, (III) working the day shift during the data collection period. Exclusion criteria: (I) History of major head trauma or work accident, (II) diagnosis of schizophrenia or bipolar disorder in first-degree relatives, (III) diagnosis of any psychiatric/neurological disorder, (IV) use of pharmacological agents affecting cognitive functions (psychostimulants, antidepressants, anxiolytics, etc.) within the last year, (V) excessive alcohol consumption within the last month, (VI) history of substance use within the last six months. Participants in the control group were selected from occupations that worked shifts, including night shifts, during the study period. The socio-demographic characteristics and statistical summaries of psychometric test scores of the participant groups are listed in Table 1.

**Table 1:** Sociodemographic characteristics and statistical summaries of psychometric test scores of the participant groups

Variables	Underground miners	Control group	Test statistics	p-value
Age	36.52 ± 5.08	35.42 ± 5.04	0.673	0.505^*^
Education level (yr)	12.63 ± 2.00	12.63 ± 2.00	0.000	1.000
Working Duration (yr)	10.84 ± 4.76	10.68 ± 5.98	−0.191	0.849
Trail Making Test-A	34.78 ± 8.11	28.58 ± 5.92	−2.434	0.015
Trail Making Test-B	87.22 ± 28.63	62.63 ± 14.90	−2.890	0.004
Immediate memory	5.31 ± 1.60	7.16 ± 1.34	−3.329	0.001
Recall	10.95 ± 2.01	13.26 ± 1.59	−3.375	0.001

^*^Independent sample t-test was applied. Mann–Whitney U test was applied

### Examining ICA and ASR

Independent Component Analysis (ICA) was performed on each preprocessed 6-s segment using the FastICA algorithm included by *FastICA toolbox v2.5* (Hyvärinen et al. 1999; Gävert et al. 2005), implemented in the *NIRS-KIT toolbox***.** For each EEG segment, the algorithm initially decomposed the data into several independent components equal to the number of recording channels. Potential artifact-related components were then automatically identified using a kurtosis threshold of 1.25 and an amplitude threshold of 4 standard deviations. These components were excluded, and the remaining independent components were back-projected to the sensor space to reconstruct the cleaned EEG signals.

In practice, we observed that none of the channels had to be rejected, so the reconstruction always retained the complete set of components equal to the number of EEG channels. This is likely due to the preceding preprocessing steps, which effectively attenuated muscle-related high-frequency activity and other large-amplitude artifacts prior to ICA. To mitigate high-amplitude artifacts (e.g., movement and muscle activity) without discarding entire data segments, we employed ASR according to the principles implemented in the clean_rawdata EEGLAB ver. *2025.0.0* plugin. ASR was developed by Kothe, the main developer of *BCILAB* (Kothe and Makeig 2013). Specifically, we adapted the algorithm into an independent MATLAB function for single-channel signals. ASR works by estimating a reference covariance matrix from the continuous data, performing eigendecomposition, and monitoring short sliding windows (default: 0.5 s with 50% overlap). For each window, the variance is compared against a threshold defined as the mean eigenvalue plus *cutoff* × standard deviation (here, cutoff = 5). If the variance exceeds this limit, the affected window is projected back into a lower-dimensional subspace (controlled by *maxdims*, default 0.66), thereby suppressing transient high-amplitude artifacts while preserving the underlying neural signal structure. In our analysis, EEG recordings were first segmented into 6-s epochs and preprocessed. Then, each epoch was processed independently with the ASR function, ensuring that artifact correction was applied consistently across trials. This implementation follows the same statistical rationale as the canonical *EEGLAB*-based ASR approach and has been shown to effectively reduce non-neural contamination in EEG (Chang et al. 2018, 2020).

### Connectivity measures

Graph theory-based functional brain connectivity analysis provides a robust framework for understanding the topological properties of brain networks through various quantitative metrics so called Modularity (Q), Global Efficiency (GE), Local Efficiency (LE), Clustering Coefficient (CC), Transitivity (T) and Assortativity (R) (Bullmore and Sporns 2012; Rubinov & Sporns 2010). Q quantifies the network's tendency to divide into modules (clusters with dense internal connections and sparse inter-module connections), where high modularity signifies functional segregation and specialized information processing. GE quantifies the overall efficiency of information transfer across the network, where high GE, associated with shorter path lengths, reflects rapid and integrated information exchange between brain regions, indicative of functional integration. LE measures the efficiency of connections among a node's neighbors, quantifying local information processing capacity and thus representing functional segregation. The CC measures the degree to which a node's neighbors are interconnected; a high clustering coefficient indicates local cliquishness and segregation, reflecting the density of local functional connections. T measures the proportion of triadic structures (triangles) in the network, reflecting local connectivity density and thus segregation. R assesses the tendency of nodes to connect with others of similar degree; positive assortativity suggests network resilience, while negative assortativity may indicate vulnerability. These metrics collectively provide critical insights into the functional organization of brain networks at both local and global levels, with applications in the study of neurological disorders and cognitive performance.

In detail, functional integration refers to the brain's ability to coordinate and combine information across distributed regions to produce unified cognitive or behavioral outcomes. This process relies on the efficient communication between distant brain areas, often facilitated by long-range connections in neural networks. Mechanistically, functional integration is supported by synchronized neural activity, as measured by EEG or fMRI, and is reflected in metrics such as GE in graph-theoretic analyses. High functional integration enables the brain to perform complex tasks, such as decision-making or perception, by integrating sensory, motor, and cognitive information. For instance, during a cognitive task, the prefrontal cortex may integrate inputs from sensory areas to guide behavior, relying on coherent oscillatory activity across frequency bands. This concept is critical in understanding how the brain achieves holistic processing despite its distributed architecture (Friston 2011).

Functional segregation describes the brain's capacity to process information in specialized, localized regions, or modules, enabling distinct functional roles. This principle is rooted in the brain's modular organization, in which specific areas, such as the visual cortex and language areas, are dedicated to particular tasks. Segregation is quantified by metrics like LE, CC, or Q in graph theory, which highlight dense local connectivity within functional units. Mechanistically, segregation arises from localized neural circuits with high within-region connectivity, often driven by short-range synaptic interactions. For example, the primary visual cortex processes specific visual features (e.g., orientation) independently before integrating them with other regions. Functional segregation ensures efficient, specialized processing, which is essential for tasks requiring distinct computational roles (Bullmore & Sporns 2012; Rubinov & Sporns 2010).

Network resilience refers to the brain's ability to maintain functionality in the face of disruptions, such as lesions, noise, or pathological conditions. This property is linked to the topological structure of brain networks, particularly their redundancy and connectivity patterns. Resilient networks often exhibit high R (where nodes of similar degree connect) or robust modular structures, allowing the brain to reroute information or compensate for damage. Mechanistically, resilience is supported by neuroplasticity and the dynamic reconfiguration of neural connections, enabling the brain to adapt to challenges such as injury or disease. For example, after a stroke, resilient brain networks may reorganize to restore function by leveraging alternative pathways. This concept is crucial for understanding recovery from brain injuries and the robustness of neural systems (Bullmore & Sporns 2012).

#### Directed transfer function (DTF)

DTF is a multivariate connectivity estimator based on the Multivariate Autoregressive (MVAR) model, rooted in Granger causality principles. It quantifies directed information flow between channels as a function of frequency, capturing causal interactions in brain networks. The MVAR model represents EEG signals as a linear combination of past values plus noise, transforming the time domain into the frequency domain via a Z-transform to derive a transfer matrix. For the DTF computation, each EEG epoch was modeled using a MVAR framework, and the model order (*p*) was optimized for every epoch. Specifically, we evaluated candidate orders within a predefined range (*p* = 1–30) by fitting an autoregressive model of order *p* to the multichannel data using the arx routine from MATLAB’s System Identification Toolbox. For each candidate order, we computed the residual covariance matrix and derived the corresponding log-likelihood of the model fit. The optimal order was then selected as the one that minimized a chosen information criterion, namely the Bayesian Information Criterion (BIC). In addition, the stability of the estimated MVAR coefficients was checked using the eigenvalue criterion, and unstable solutions were penalized. The order yielding the lowest valid criterion value across the tested range was chosen as the best model order for that epoch. DTF is calculated as the normalized magnitude of this transfer matrix, specifically the element ${H}_{ij}(f)$ which represents the directed influence from channel $j$ at frequency $f$. The formula is:

$$DTF_{j \to i} \left( f \right) = \frac{{\left| {H_{ij} \left( f \right) } \right|^{2} }}{{\mathop \sum \nolimits_{k} \left| {H_{ij} \left( f \right) } \right|^{2} }}$$

This normalization ensures that DTF values range between 0 and 1, reflecting the relative strength of directed connectivity. DTF accounts for multivariate interactions, distinguishing direct from indirect connections, and is particularly effective for identifying frequency-specific causal relationships. DTF is inherently robust to volume conduction because it relies on phase differences between channels, which are unaffected by the instantaneous propagation of electromagnetic fields (volume conduction). Volume conduction introduces zero-phase-lag correlations, which do not contribute to DTF estimates, as DTF is non-zero only when a phase difference exists. Simulations have demonstrated that adding a constant-phase signal (e.g., a 20 Hz sinusoid) to EEG data does not alter DTF results, confirming its insensitivity to volume conduction. This robustness eliminates the need for preprocessing steps like source projection or Independent Component Analysis (ICA), which can introduce errors if misapplied (Jung et al. 2000; Kaminski et al. 2014). However, ICA is applied to noisy EEG segments.

#### Imaginary Part of Coherence (iCOH)

iCOH measures functional connectivity by focusing on the imaginary component of the coherence function, which quantifies the phase relationship between two signals. Coherence is defined as the normalized cross-spectral density:

$$COH_{ij} \left( f \right) = \frac{{S_{ij} \left( f \right)}}{{\sqrt {S_{ii} \left( f \right)S_{jj} \left( f \right)} }}$$

where ${S}_{ij}(f)$ is the cross-spectral density between channels $i$ and$j$, ${S}_{ii}(f)$ and ${S}_{jj}(f)$ refer the auto-spectral densities of each channel. The imaginary part of${COH}_{ij}\left(f\right)$, isolates interactions with non-zero phase lags, as it is maximal when the phase difference is $\pm \pi /2$ and zero when the phase difference is 0 or $\pi$. iCOH thus captures true neural interactions while discarding instantaneous correlations. However, iCOH may underestimate connectivity when true interactions occur at zero or $\pi$-phase differences, as it ignores the real part of coherence, which may contain relevant information (Sanchez et al. 2018).

iCOH is designed to be robust against volume conduction because it exclusively uses the imaginary component of coherence, which is unaffected by zero-phase-lag effects caused by volume conduction. Since volume conduction results from the instantaneous spread of electric fields, it produces correlations with no phase lag, which are filtered out by iCOH. However, its reliance on the imaginary part may cause it to miss true interactions with zero or π-phase lags, potentially reducing sensitivity in certain scenarios. Despite this, iCOH provides a reliable estimate of functional connectivity in sensor-space EEG/MEG, as demonstrated in studies comparing it to other methods (Sanchez et al. 2018).

***Weighted phase lag index (wPLI)***

wPLI is an extension of the Phase Lag Index (PLI), which measures the asymmetry in the distribution of phase differences between two signals. PLI is defined as:

$$PLI_{ij} = \left| {sign\left( {\Phi_{ij} \left( t \right)} \right)} \right|$$

where ${\Phi }_{ij}(t)$ is the phase difference between signals $i$ and $j$ at time $t$, sign refers the signum function. wPLI improves upon PLI by weighting phase differences by the magnitude of the imaginary part of the cross-spectral density, emphasizing larger phase lags:

$$wPLI_{ij} = \frac{{\left| {\left| {{\mathrm{Im}} \left( {S_{ij} } \right)} \right|sign\left( {{\mathrm{Im}} \left( {S_{ij} } \right)} \right)} \right|}}{{\left| {{\mathrm{Im}} \left( {S_{ij} } \right)} \right|}}$$

This weighting reduces the influence of small phase differences near zero, enhancing robustness against noise and volume conduction. wPLI values range from 0 to 1, with higher values indicating stronger phase synchronization. The debiased wPLI estimator further improves accuracy by correcting for sample-size bias (Vinck et al. 2011).

wPLI is highly effective at mitigating volume conduction effects because it focuses on phase differences and weights them by the imaginary part of the cross-spectrum, which is insensitive to zero-phase-lag correlations. By downweighting phase differences near zero, wPLI reduces false positives caused by volume conduction or common-reference artifacts. Studies have shown that wPLI provides clearer topographic connectivity maps compared to coherence, particularly in the alpha band, due to its robustness against volume conduction and noise (Ortiz et al.^2012^).

#### Estimation of graph theoretical indices

**Fig. 1:**
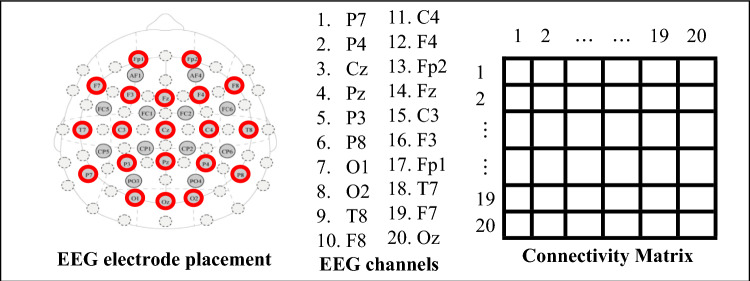
EEG electrode placement, recording channel numbers used in the construction of the connectivity matrix, and their correspondence with matrix elements

 For each participant, in a given state (EO vs EC) and within a specified EEG frequency band, inter-hemispheric connectivity levels obtained from each 6-s segment of the 20-channel surface EEG recordings, using a connectivity method (DTF, iCOH, wPLI), were organized into a connectivity matrix, as illustrated in Fig. 1.

The connectivity matrices derived from the DTF, iCOH, and wPLI measures, were converted into binary adjacency matrices prior to computing network metrics. Because DTF estimates directed (asymmetric) connections, the resulting adjacency matrix is not symmetric. A proportional threshold corresponding to 60% of the maximum connection weight was applied to derive binary adjacency matrices, ensuring the preservation of the strongest functional connections while maintaining network comparability across subjects in recognizing emotional states identified by statistical and effective connectivity estimations (Kılıç & Aydın, 2022; Aydın & Onbaşı, 2024). In the present study, we applied a minimum-spanning-tree (MST) based thresholding strategy to select the most informative connections while preserving the global structure of the network. Specifically, the weighted directed DTF matrix was first inverted so that the MATLAB minspantree function could identify the tree of strongest connections. The MST ensured that all nodes remained connected using the minimal set of highest-weight edges. We then added the strongest remaining non-MST edges until a fixed target network density (e.g., 15% of all possible edges) was reached, after which the matrix was binarized for graph-theoretic computations. For the wPLI and iCOH measures, which produce undirected (symmetric) connectivity matrices, thresholding was simpler because edge directionality was not considered. After symmetrizing the matrices, we applied a proportional threshold, retaining only the top-strength connections up to a predefined density level, followed by binarization. To achieve this, all non-MST connections were sorted in descending order of strength, and the strongest edges were added until the target density was reached. The threshold for inclusion was defined by the weakest edge that met this density criterion. In the final step, the combined MST and additional strong connections were binarized, yielding a fully connected, comparable binary adjacency matrix that retained only the strongest links. This two-tiered approach ensured that, for DTF, the network remained fully connected and directionality was preserved via MST-guided thresholding, whereas for wPLI/iCOH, a standard density-controlled threshold on the symmetric matrix was sufficient. On these binary network, six different connectivity indices (Q, GE, LE, CC, T, R) were calculated using functions from the Brain Connectivity Toolbox (BCT) (Rubinov & Sorns, 2010; 2011).

For directed networks, modularity (Q) is defined as,

$$Q = \frac{1}{m}\mathop \sum \limits_{ij} \left( {A_{ij} - \frac{{k_{i}^{out} .k_{j}^{in} }}{m}} \right)\delta \left( {c_{i} ,c_{j} } \right)$$

where ${A}_{ij}$​ is the adjacency matrix, ${k}_{i}^{out}$ and ${k}_{j}^{in}$​ denote the out-degree and in-degree of nodes $i$ and $j$, $m$ is the total number of edges, and $\delta \left({c}_{i},{c}_{j}\right)$ equals 1 when nodes belong to the same community.

Global Efficiency (GE) is defined by,

$$GE = \frac{1}{{N\left( {N - 1} \right)}}\mathop \sum \limits_{i \ne j} \frac{1}{{d_{ij} }}$$

where ${d}_{ij}$​ is the shortest directed path length from node $i$ to node $j$. If no directed path exists, the term is treated as zero.

Local Efficiency (LE) in directed networks is defined as the average global efficiency computed on each node’s directed neighborhood subgraph in form,

$$LE = \frac{1}{N}\mathop \sum \limits_{i} GE\left( {G_{i} } \right)$$

where ${G}_{i}$ is the subgraph composed of neighbors of node $i$, preserving directionality.

Clustering Coefficient (CC) is obtained as the averaged network-level coefficients calculated in form,

$$CC = \frac{{t_{i} }}{{k_{i}^{tot} \left( {k_{i}^{tot} - 1} \right) - 2k_{i}^{ \leftrightarrow } }}$$

where ${t}_{i}$ is the number of directed triangles around node $i$, ${k}_{i}^{tot}={k}_{i}^{out}+{k}_{i}^{in}$, and ${k}_{i}^{\leftrightarrow }$ is the number of reciprocal (bidirectional) connections.

For directed grpahs, Transitivity (T) is defined as,

$${\mathrm{T}} = \frac{{\text{number of directed triangles}}}{{{\text{number of connected directed triplets}} }}$$

Assortativity (R) in directed networks measures the Pearson correlation between degrees of connected node pairs. Directed assortativity can be defined based on combinations of in-degree and out-degree (e.g., out–in degree correlation). In this study, R was computed using the directed degree correlation implemented in the BCT.

Importantly, the target density was kept identical across all participants and connectivity measures to ensure direct comparability between subjects and groups. Because graph-theoretical metrics are known to be sensitive to variations in network density, enforcing a fixed proportional density prior to binarization minimized potential density-driven bias in subsequent analyses.

#### Group comparisons through linear mixed effect models

Recent advances in EEG analysis and network neuroscience emphasize that averaging over trials or epochs may discard valuable within-subject variability, and instead recommend using Linear Mixed Effects models (LMEs) to properly model repeated observations. For example, in a comparative framework, the researchers illustrate how LMEs often outperform classical ANOVA in EEG analysis by better accounting for intra-subject variance and reducing false positives (Frömer et al. 2018). Moreover, other works explicitly recommend replacing subject-wise averaging with LMEs when feasible, pointing to LMEs' increased sensitivity and robustness under realistic noise conditions (e.g., unequal trial counts, missing data) compared with ANOVA approaches (Riha et al. 2020). In a more recent study, Heise et al. (2022) argue that traditional averaging of epochs can bias results when trial counts differ across participants, and demonstrate that LME yields more accurate, unbiased estimates even with missing or sparse data. Thus, by adopting LME in our analysis, we align with best practices in the neurophysiology and EEG literature, modeling both fixed group differences and random subject effects while fully utilizing the epoch-level data.

In this study, to account for the repeated-measures structure of the data (40 epochs per participant across both EO and EC states) and to improve on the simple group-wise t-tests originally reported, we fitted Linear Mixed Effects models (LMEs) for each graph-theoretic index across all frequency-band combinations and eye states. Therefore, for each LME, all single epoch estimations from both groups (*Controls* vs *Miners*) were first arranged in a long format table including the variables: Value: graph metric score of a given participant at a given epoch, Group: categorical fixed factor with two levels (*Control* as the reference level and *Miners* as the second group), Subject: participant identifier to model subject-specific variability, Epoch**:** repeated-measurement index (1–40). The primary model specification was in form,

$$Value_{ij} = \beta_{0} + \beta_{1} + Group_{i} + u_{i} + \varepsilon_{ij}$$

where ${\beta }_{1}$ captures the mean difference between groups, ${u}_{i}\sim N\left(0,{\sigma }_{u}^{2}\right)$ is the random intercept for $subject i$, and ${\varepsilon }_{i}\sim N\left(0,{\sigma }^{2}\right)$ is the residual error for $epoch j$ within $subject i$. LMEs were estimated using MATLAB’s fitlme function with syntax: Value ~ Group + (1 | Subject). This approach appropriately accounts for the non-independence of epoch-wise observations within each participant and relaxes the normality assumption required by ordinary *t*-tests.

For each state–band–index combination, the β₁ coefficient represents the estimated mean difference between the *Miners* group and the *Control* group (reference). A negative β₁ indicates lower average values in *Miners* compared with *Control*, while a positive β₁ indicates higher average values in *Miners*. The associated *p*-value tests the null hypothesis that β₁ = 0 (no group effect). This LME framework allowed us to test group differences while fully exploiting the repeated-epoch structure of the dataset and provided more reliable inference than simple independent-samples tests.

## Results

In tests, surface EEG data, recorded with 20 electrodes during EO and EC resting states, were initially segmented into non-overlapping 6-s epochs. A notch filter, a butterworth filter and a band-pass filter were applied to each segment. In the secondary processing stage, DTF, iCOH and wPLI methods were applied to EEG frequency bands after preprocessing with ASR or ICA. Six graph-theoretical connectivity indices (Q, GE, LE, CC, T, R) were derived.

**Fig. 2:**
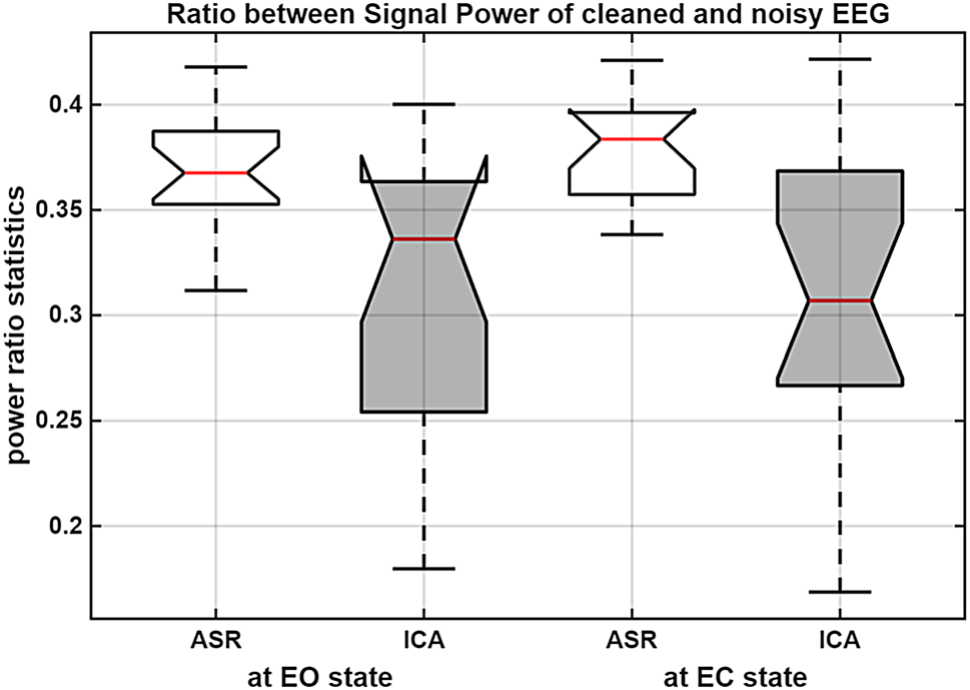
Grand averaged signal power ratios in the control group, computed across 19 participants and 20 EEG channels, using 40 non-overlapping 6-s epochs. Results were shown separately for EO and EC states, comparing two preprocessing approaches (ASR and ICA)

As a first step, to ensure that the connectivity indices estimated from the resting-state EEG recordings were stable and reliable, we computed the ICCs for each method combination. For each participant in both groups (19 individuals per group), 4-min EO (excluding the first 10 s) and 4-min EC (starting 10 s after eye closure) resting-state EEG segments were analyzed. Each condition was divided into 40 non-overlapping 6-s epochs, and connectivity indices were estimated for each epoch. The ICCs for all method combinations were then calculated separately for each group. The results obtained for the miners are summarized in Table 2, whereas those for the control group are provided in Table 3. To evaluate which of the two preprocessing approaches yielded more stable and favorable estimates, we primarily relied on ICCs. In addition, within the control group, we computed signal power ratios by dividing the power of the preprocessed signal by the power of the corresponding unprocessed (noisy) signal for each individual and each non-overlapping short epoch. These power ratios were then averaged across EEG channels and subjects, and the box plots of the grand-averaged results are shown in Fig. 2. As illustrated in Fig. 2, in both EO and EC states, the ASR preprocessing approach resulted in higher SNR values than ICA. Moreover, the narrower box widths indicate reduced variability across participants and EEG recording channels, suggesting that ASR yields more consistent and robust enhancement of signal quality.

When considering the ICCs listed in Table 2, in the miners group, the DTF method with ICA integration yielded good to predominantly excellent ICCs across all indices in both EO and EC states within the gamma and full bands, and good to excellent ICCs across all indices in the beta band at the EC state. When DTF was combined with ASR, it consistently provided *excellent* ICC values, along with only a few *good* ones, across all frequency bands, all indices, and both EO and EC states. The other two approaches did not demonstrate comparable reliability. Specifically, in the delta band, iCOH failed to yield any useful/acceptable ICC values at either EO or EC. In contrast, with ASR integration it achieved *excellent* ICC values across all indices at EC state. Furthermore, across the beta, gamma, and full bands, ASR integration again produced excellent ICC values using iCOH. Regarding the wPLI method, ICA integration produced acceptable ICC values for only Q, GE, and R indices in the gamma and full bands at both EO and EC states. However, wPLI with ASR integration, only a few indices and frequency bands provided acceptable ICC values, making this the least reliable approach overall.

When considering the ICC values listed in Table 3, the DTF method with both ICA and ASR integration yielded *excellent* ICC values across all indices in the beta, gamma, and full bands under both EO and EC states. The iCOH method, while not providing acceptable ICC values with ICA integration, achieved *good* to *excellent* ICC values in the delta, beta, gamma, and full bands under both EO and EC when combined with ASR integration. For the wPLI method, ICA integration produced *good* to *excellent* ICC values in the gamma band. In contrast, ASR integration yielded predominantly *good*—along with some *excellent*—ICC values in the gamma and full bands under both EO and EC states, and in the beta band under the EC state.

When Table 2 and Table 3 are considered together, it is evident that ICA integration yielded substantially more negative ICCs in both miners and controls compared to ASR integration. Moreover, the control group demonstrated more stable estimations overall. Importantly, in both groups, the most consistent results were obtained with the DTF–ASR combination. Therefore, statistical differences between groups were investigated using LMEs for the particular combination of DTF and ASR that yielded the most reliable estimates. Table 4 presents the outcomes of the LMEs examining group differences between miners and controls for each graph-theoretical index that was estimated using the DTF and ASR approaches. Only indices that reached acceptable reliability (ICC ≥ 0.60) were included in these analyses. The statistical criterion for significance was the false discovery rate (FDR) corrected p-values (*p*_*c*_) across all tests (two-tailed, α = 0.05). For each index and frequency band, the fixed-effect estimate (β_1_) indicates the mean difference between the two groups, with controls serving as the reference. 95% confidence intervals are provided for β_1_, and positive β_1_ values indicate higher values in the miners group than in controls.

**Table 2:** ICCs in Miners (negative values are given in italics and underlined form where the values were highlighted as *good* for 0.60 ≤ ICC < 0.75, and *excellent* for ICC ≥ 0.75)

		**ICA**						**ASR**					
		**DTF**		**iCOH**		**wPLI**		**DTF**		**iCOH**		**wPLI**	
		EO	EC	EO	EC	EO	EC	EO	EC	EO	EC	EO	EC
δ	Q	**0.85**	0.53	0.26	0.32	*0.26*	0.50	**0.81**	**0.80**	**0.90**	**0.93**	**0.61**	0.54
	GE	**0.93**	0.53	0.53	0.16	*0.24*	0.53	**0.76**	**0.66**	**0.90**	**0.94**	0.31	0.09
	LE	0.54	0.23	0.42	*0.02*	0.07	0.27	**0.92**	**0.91**	**0.93**	**0.92**	0.35	0.08
	CC	0.38	0.27	0.43	0.01	0.09	0.18	**0.92**	**0.90**	**0.93**	**0.91**	0.42	0.30
	T	0.57	0.39	0.46	*0.37*	*0.17*	0.51	**0.87**	**0.88**	**0.92**	**0.91**	0.48	0.03
	R	**0.87**	**0.63**	0.54	*0.05*	0.18	**0.67**	**0.93**	**0.94**	**0.95**	**0.92**	**0.79**	**0.79**
θ	Q	0.54	0.58	0.21	0.44	0.28	0.34	**0.82**	**0.72**	**0.78**	**0.70**	0.31	0.43
	GE	**0.64**	0.51	*0.03*	0.59	0.02	0.35	**0.81**	**0.76**	**0.69**	0.58	0.37	0.56
	LE	0.51	0.39	0.09	0.41	0.11	0.04	**0.92**	**0.91**	**0.63**	0.50	0.50	0.48
	CC	0.53	0.47	0.15	0.35	0.10	0.07	**0.92**	**0.91**	*0.41*	0.48	**0.65**	0.43
	T	**0.60**	0.41	0.07	0.23	0.05	*0.44*	**0.84**	**0.87**	**0.67**	**0.66**	0.31	0.18
	R	0.55	0.42	0.18	0.52	*0.88*	0.24	**0.94**	**0.92**	**0.90**	**0.86**	**0.79**	**0.79**
α	Q	0.57	0.71	0.34	0.56	*0.24*	0.16	**0.74**	**0.90**	**0.81**	**0.89**	0.48	0.50
	GE	**0.73**	**0.61**	**0.68**	**0.75**	*0.15*	0.46	**0.85**	**0.86**	**0.68**	**0.72**	0.36	0.54
	LE	0.22	0.40	0.20	0.54	0.19	0.12	**0.90**	**0.92**	*0.02*	0.19	0.49	0.56
	CC	0.32	0.46	0.20	0.53	0.08	0.03	**0.88**	**0.91**	0.53	0.43	**0.62**	0.59
	T	0.19	**0.61**	***0.64***	0.24	0.13	0.37	**0.88**	**0.90**	0.36	**0.67**	0.53	0.43
	R	0.02	*0.01*	**0.74**	**0.76**	*0.05*	0.40	**0.95**	**0.94**	0.58	**0.70**	**0.79**	**0.79**
β	Q	0.58	**0.69**	*0.03*	**0.71**	0.52	**0.74**	**0.81**	**0.90**	**0.91**	**0.95**	*0.20*	0.26
	GE	**0.87**	**0.82**	0.25	**0.74**	0.47	**0.84**	**0.87**	**0.88**	**0.81**	**0.91**	0.53	**0.64**
	LE	0.42	**0.80**	**0.60**	**0.60**	0.32	0.45	**0.79**	**0.93**	**0.71**	**0.63**	0.58	**0.66**
	CC	0.25	**0.81**	**0.62**	**0.62**	0.31	0.45	**0.76**	**0.93**	**0.82**	**0.75**	0.59	0.54
	T	0.44	**0.82**	0.40	0.44	0.54	**0.68**	**0.84**	**0.93**	**0.76**	**0.68**	**0.64**	**0.65**
	R	**0.71**	**0.71**	**0.65**	**0.81**	0.52	**0.84**	**0.93**	**0.95**	**0.80**	**0.86**	**0.79**	**0.79**
γ	Q	**0.85**	**0.83**	**0.71**	**0.88**	**0.75**	**0.93**	**0.77**	**0.87**	**0.95**	**0.96**	*0.25*	0.50
	GE	**0.85**	**0.70**	**0.78**	**0.81**	**0.83**	**0.91**	**0.81**	**0.88**	**0.94**	**0.94**	0.55	**0.71**
	LE	**0.83**	**0.88**	0.42	0.55	*0.18*	**0.74**	**0.77**	**0.93**	**0.83**	**0.81**	**0.62**	**0.70**
	CC	**0.78**	**0.87**	0.44	0.54	*0.27*	**0.73**	**0.74**	**0.93**	**0.81**	**0.83**	**0.73**	**0.67**
	T	**0.91**	**0.90**	0.25	**0.71**	0.57	**0.83**	**0.80**	**0.89**	**0.79**	**0.61**	**0.65**	**0.72**
	R	**0.89**	**0.88**	**0.78**	**0.85**	**0.82**	**0.92**	**0.88**	**0.93**	**0.79**	**0.79**	**0.79**	**0.79**
f	Q	**0.80**	**0.79**	**0.91**	**0.93**	**0.71**	**0.77**	**0.80**	**0.82**	**0.96**	**0.95**	0.48	0.35
	GE	**0.90**	**0.81**	**0.87**	**0.89**	**0.73**	**0.81**	**0.88**	**0.86**	**0.93**	**0.91**	0.59	0.45
	LE	**0.78**	**0.71**	0.45	**0.81**	0.44	0.53	**0.78**	**0.92**	**0.79**	**0.79**	**0.67**	0.43
	CC	**0.69**	**0.70**	0.44	**0.81**	0.43	0.54	**0.75**	**0.92**	**0.74**	**0.77**	0.36	0.52
	T	**0.77**	**0.82**	**0.63**	**0.76**	0.57	**0.62**	**0.78**	**0.90**	**0.80**	**0.77**	**0.68**	**0.64**
	R	**0.86**	**0.78**	**0.81**	**0.90**	**0.74**	**0.75**	**0.89**	**0.94**	**0.79**	**0.61**	**0.79**	**0.79**

**Table 3:** ICCs in Controls (negative values are given in italics and underlined form where the values were highlighted as *good* for 0.60 ≤ ICC < 0.75, and *excellent* for ICC ≥ 0.75)

		ICA						ASR					
		DTF		iCOH		wPLI		DTF		iCOH		wPLI	
		EO	EC	EO	EC	EO	EC	EO	EC	EO	EC	EO	EC
δ	Q	**0.80**	**0.67**	0.53	0.12	0.49	0.44	**0.89**	**0.89**	**0.67**	**0.80**	**0.66**	0.33
	GE	**0.88**	**0.68**	0.36	0.46	**0.60**	**0.61**	**0.87**	**0.81**	**0.86**	**0.88**	0.57	0.31
	LE	**0.79**	**0.71**	0.45	*0.38*	*0.01*	0.59	**0.94**	**0.91**	**0.95**	**0.95**	0.50	0.40
	CC	**0.75**	**0.70**	0.45	*0.47*	*0.02*	0.59	**0.94**	**0.91**	**0.94**	**0.94**	0.55	0.34
	T	**0.82**	**0.77**	0.57	0.48	0.45	**0.69**	**0.94**	**0.90**	**0.96**	**0.95**	0.55	0.31
	R	**0.81**	**0.67**	**0.66**	0.58	0.59	**0.62**	**0.94**	**0.91**	**0.96**	**0.96**	**0.79**	**0.79**
θ	Q	0.26	0.29	*0.09*	0.55	0.36	0.25	**0.85**	**0.84**	**0.68**	**0.75**	**0.66**	0.47
	GE	**0.61**	**0.66**	*0.60*	**0.60**	0.39	0.41	**0.88**	**0.80**	**0.61**	0.59	0.57	0.36
	LE	0.07	0.30	0.11	0.28	*0.44*	*0.15*	**0.94**	**0.88**	**0.72**	**0.62**	0.44	0.48
	CC	0.05	0.31	0.19	0.32	*0.49*	*0.20*	**0.94**	**0.87**	0.53	0.56	**0.76**	0.55
	T	0.23	0.46	*0.06*	0.32	0.00	0.49	**0.94**	**0.86**	**0.83**	**0.82**	0.59	0.48
	R	**0.61**	0.47	0.38	**0.70**	0.38	0.39	**0.93**	**0.88**	**0.94**	**0.90**	**0.79**	**0.79**
α	Q	0.52	**0.68**	**0.68**	0.58	0.05	0.29	**0.81**	**0.89**	**0.85**	**0.89**	**0.66**	0.33
	GE	0.51	**0.65**	**0.73**	**0.81**	0.33	**0.61**	**0.89**	**0.90**	**0.74**	**0.81**	**0.66**	0.24
	LE	**0.68**	**0.79**	0.05	0.30	*0.12*	0.37	**0.94**	**0.89**	0.48	0.30	0.56	0.44
	CC	**0.72**	**0.81**	0.09	0.33	*0.18*	0.37	**0.93**	**0.88**	0.56	0.51	**0.82**	0.55
	T	0.41	0.47	0.25	0.45	0.23	0.54	**0.92**	**0.87**	**0.64**	**0.76**	**0.66**	0.59
	R	0.23	0.46	**0.77**	**0.80**	0.55	0.54	**0.93**	**0.91**	**0.82**	**0.83**	**0.79**	**0.79**
β	Q	**0.77**	**0.90**	0.01	0.40	0.56	0.54	**0.81**	**0.89**	**0.89**	**0.92**	0.26	**0.61**
	GE	**0.81**	**0.86**	0.38	**0.77**	**0.70**	**0.75**	**0.83**	**0.92**	**0.81**	**0.89**	0.42	**0.75**
	LE	**0.81**	**0.90**	0.04	0.59	0.36	0.35	**0.82**	**0.94**	**0.79**	**0.78**	0.39	**0.78**
	CC	**0.76**	**0.90**	0.05	0.58	0.38	0.31	**0.80**	**0.94**	**0.77**	**0.66**	**0.83**	**0.71**
	T	**0.86**	**0.94**	0.31	**0.71**	0.39	**0.62**	**0.78**	**0.94**	**0.81**	**0.82**	0.64	**0.82**
	R	**0.87**	**0.76**	0.42	**0.75**	**0.73**	**0.67**	**0.92**	**0.94**	0.59	**0.71**	**0.79**	**0.79**
γ	Q	**0.94**	**0.95**	**0.76**	**0.66**	**0.78**	**0.87**	**0.75**	**0.92**	**0.93**	**0.89**	**0.66**	**0.60**
	GE	**0.85**	**0.88**	**0.83**	**0.86**	**0.80**	**0.89**	**0.84**	**0.93**	**0.82**	**0.80**	**0.62**	**0.72**
	LE	**0.93**	**0.95**	0.47	0.41	**0.70**	**0.60**	**0.84**	**0.92**	**0.89**	**0.68**	**0.61**	0.80
	CC	**0.93**	**0.95**	0.49	0.45	**0.69**	0.57	**0.83**	**0.92**	**0.82**	**0.72**	0.84	**0.73**
	T	**0.95**	**0.97**	0.49	0.56	**0.87**	**0.86**	**0.78**	**0.93**	**0.85**	**0.75**	**0.80**	**0.83**
	R	**0.94**	**0.91**	**0.80**	**0.85**	**0.81**	**0.85**	**0.89**	**0.93**	**0.69**	**0.82**	**0.79**	**0.79**
f	Q	**0.87**	**0.93**	0.21	**0.67**	0.56	**0.68**	**0.77**	**0.93**	**0.93**	**0.88**	**0.67**	0.18
	GE	**0.86**	**0.88**	0.55	**0.79**	0.51	**0.81**	**0.80**	**0.92**	**0.78**	**0.75**	**0.62**	**0.61**
	LE	**0.90**	**0.91**	0.53	**0.74**	**0.61**	0.52	**0.85**	**0.93**	**0.87**	**0.75**	**0.66**	**0.70**
	CC	**0.91**	**0.91**	0.53	**0.74**	**0.62**	0.46	**0.84**	**0.93**	**0.82**	**0.75**	**0.81**	**0.74**
	T	**0.90**	**0.93**	0.33	**0.63**	**0.60**	**0.80**	**0.79**	**0.94**	**0.81**	**0.84**	**0.76**	**0.73**
	R	**0.87**	**0.86**	0.62	**0.80**	0.48	**0.82**	**0.92**	**0.94**	0.52	**0.78**	**0.79**	**0.79**

As listed in Table 4, significant group differences emerged consistently for LE, CC, and T across all frequency bands under the EC state, with miners showing lower values. The assortativity index, R, also differed between groups in the EC state within the alpha, beta, gamma, and full bands. Moreover, the GE index revealed group differences in frequency band intervals as δ (delta), θ (theta), α (alpha), β (beta), γ (gamma), and f (full) under both EO and EC states. When Table 4 is examined, one of the most prominent findings is a group difference in GE between underground workers and the control group in EO state, favoring the control group across all frequency bands except gamma. The corresponding effect sizes ranged from medium to large (Cohen’s f^2^ = 0.3227 to 0.5583). However, the primary connectivity differences were predominantly detected in the eyes-closed condition, where effect sizes were mostly within the large effect.

**Table 4:** Results of the LMEs testing group differences for each frequency band specific graph theoretical index estimated by DTF and ASR (providing ICC ≥ 0.60), *p*_*c*_: corrected *p* values with FDR (α = 0.05), *f*^*2*^*:Cohen’s f*^*2*^, β_1_: the fixed-effect estimate, the reference is Controls with 95% confidence intervals

			**Q**	**GE**	**LE**	**CC**	**T**	**R**
EO	δ	${\beta }_{1}$	−0,0067	−0,0039	−0,0088	−0,0073	−0,0033	−0,0004
		*p*	**0,0001**	**0,0001**	**0,0042**	**0,0132**	0,0944	0,9573
		*p*_*c*_	**0,0002**	**0,0003**	**0,0099**	**0,0264**	0,1618	0,9573
		*f*^*2*^	**0.4453**	**0.4157**	**0.2217**	**0.1664**	0.0757	0.0001
	θ	${\beta }_{1}$	−0,0033	−0,0046	−0,0061	−0,0039	−0,0024	0,0005
		*p*	0,0429	**0,0000**	0,0452	0,1719	0,2218	0,9370
		*p*_*c*_	0,0793	**0,0000**	0,0813	0,2526	0,3132	0,9502
		*f*^*2*^	0.0999	**0.5539**	0.1086	0.0505	0.0404	0.0002
	α	${\beta }_{1}$	−0,0035	−0,0048	−0,0042	−0,0018	−0,0025	−0,0032
		*p*	0,0286	**0,0000**	0,1605	0,5227	0,1994	0,6311
		*p*_*c*_	0,0542	**0,0000**	0,2408	0,5973	0,2871	0,7100
		*f*^*2*^	0.1298	**0.5583**	0.0533	0.011	0.0446	0.0062
	β	${\beta }_{1}$	−0,0020	−0,0035	−0,0006	0,0008	0,0002	−0,0073
		*p*	0,2272	**0,0006**	0,8455	0,7683	0,9151	0,2880
		*p*_*c*_	0,3145	**0,0016**	0,8822	0,8257	0,9413	0,3912
		*f*^*2*^	0.0395	**0.3227**	0.001	0.0023	0.0003	0.0305
	γ	${\beta }_{1}$	−0,0024	−0,0018	0,0026	0,0037	0,0016	−0,0051
		*p*	0,1573	0,0625	0,3554	0,1557	0,3733	0,4328
		*p*_*c*_	0,2408	0,1097	0,4480	0,2408	0,4480	0,5026
		*f*^*2*^	0.0541	0.0939	0.0231	0.0545	0.0214	0.0166
	f	${\beta }_{1}$	−0,0016	−0,0044	0,0011	0,0024	0,0016	−0,0101
		*p*	0,3126	**0,0000**	0,6816	0,3465	0,3811	0,1133
		*p*_*c*_	0,4168	**0,0001**	0,7436	0,4455	0,4499	0,1854
		*f*^*2*^	0.0276	**0.4939**	0.0046	0.024	0.0207	0.0678
EC	δ	${\beta }_{1}$	−0,0070	−0,0044	−0,0082	−0,0064	−0,0068	−0,0106
		*p*	**0,0000**	**0,0000**	**0,0036**	**0,0176**	**0,0003**	0,1013
		*p*_*c*_	**0,0002**	**0,0000**	**0,0086**	**0,0342**	**0,0010**	0,1696
		*f*^*2*^	**0.4647**	**0.5439**	**0.2302**	**0.1527**	**0.3535**	0.0727
	θ	${\beta }_{1}$	−0,0052	−0,0047	−0,0100	−0,0076	−0,0068	−0,0058
		*p*	**0,0016**	**0,0000**	**0,0007**	**0,0065**	**0,0003**	0,3687
		*p*_*c*_	**0,0043**	**0,0000**	**0,0020**	**0,0141**	**0,0010**	0,4480
		*f*^*2*^	**0.2703**	**0.6419**	**0.3104**	**0.2011**	**0.3481**	0.0219
	α	${\beta }_{1}$	−0,0018	−0,0029	−0,0099	−0,0074	−0,0101	−0,0204
		*p*	0,3209	**0,0056**	**0,0018**	**0,0128**	**0,0000**	**0,0024**
		*p*_*c*_	0,4201	**0,0127**	**0,0045**	**0,0263**	**0,0000**	**0,0059**
		*f*^*2*^	0.0266	**0.2077**	**0.2657**	**0.1438**	**0.6349**	**0.2502**
	β	${\beta }_{1}$	−0,0053	−0,0010	−0,0146	−0,0137	−0,0133	−0,0276
		*p*	**0,0084**	0,3611	**0,0000**	**0,0000**	**0,0000**	**0,0001**
		*p*_*c*_	**0,0179**	0,4480	**0,0001**	**0,0001**	**0,0000**	**0,0003**
		*f*^*2*^	**0.188**	0.0226	**0.5253**	**0.5069**	**0.902**	**0.4259**
	γ	${\beta }_{1}$	−0,0029	−0,0004	−0,0124	−0,0115	−0,0106	−0,0243
		*p*	0,1297	0,6788	**0,0001**	**0,0001**	**0,0000**	**0,0003**
		*p*_*c*_	0,2075	0,7436	**0,0003**	**0,0004**	**0,0000**	**0,0010**
		*f*^*2*^	0.0621	0.0046	**0.4304**	**0.408**	**0.6992**	**0.3473**
	f	${\beta }_{1}$	−0,0102	−0,0003	−0,0189	−0,0178	−0,0152	−0,0344
		*p*	**0,0000**	0,8068	**0,0000**	**0,0000**	**0,0000**	**0,0000**
		*p*_*c*_	**0,0000**	0,8542	**0,0000**	**0,0000**	**0,0000**	**0,0000**
		*f*^*2*^	**0.7583**	0.0016	**0.9347**	**0.9137**	**1.325**	**0.6918**

*Note.* Effect sizes were interpreted according to Cohen’s f^2^ criteria: 0.02 = small effect, 0.15 = medium effect, and 0.35 = large effect

**Fig. 3:**
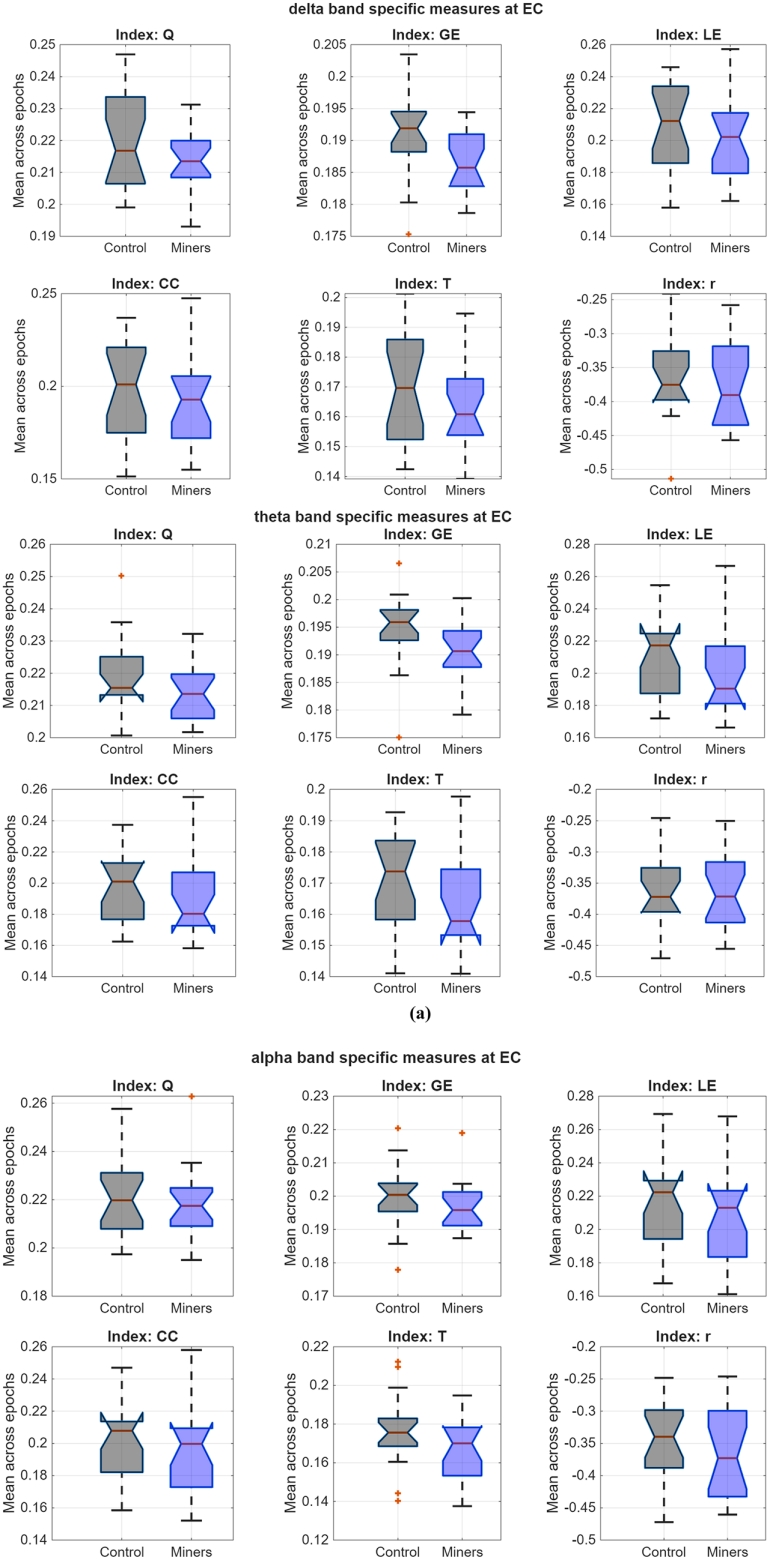

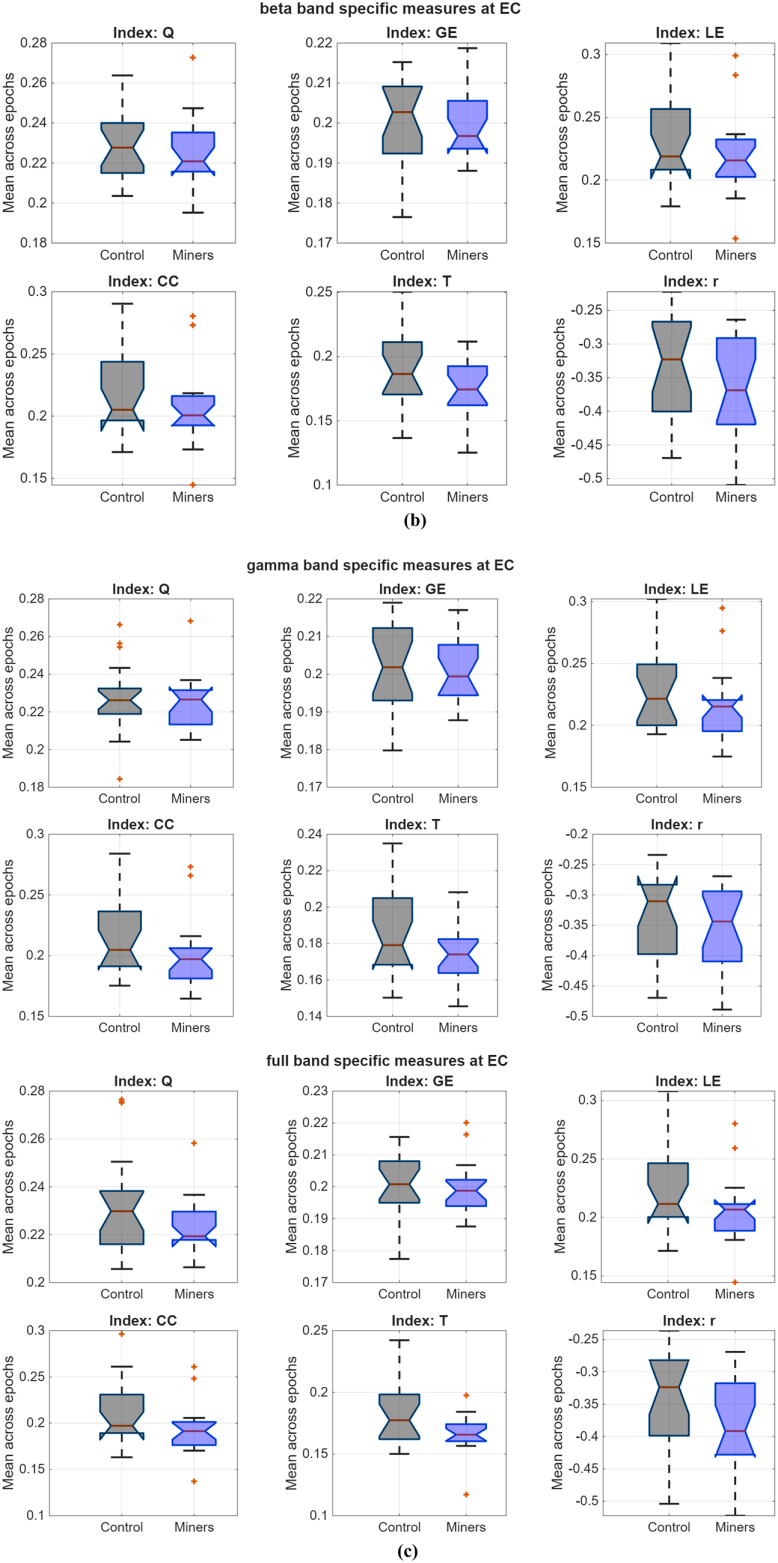
Statistical box plots of gamma subband an full-band specific connectivity indices estimated by using DTF with ASR at EC state in miners and controls

Statistical box-plots in accordance with graph theoretical indices (Q, GE, LE, CC, T, R) estimated by using DTF integrated with ASR were shown in Fig. 3 for all frequency bands. Regarding this figure, the Modularity (Q) medians of miners were considered as similar to those of controls, however, the miners’ mean values were lower than controls for all other indices in almost each sub-band. In particular, there was no outlier in lower sub-bands (delta, theta), while there were a few outliers in most indices except resilience index, R in higher sub-bands (alpha-full).

In order to assess the relationship between frequency band specific EEG-derived network indices and psychometric scores, we computed Pearson correlation coefficients in MATLAB for each subject after averaging 40 trials per individual for each index and band. Table 5 reports only those correlations that remained significant after applying the Benjamini Hochberg FDR correction (adjusted *p* ≤ 0.05). Multiple-comparison correction was performed using the Benjamini–Hochberg FDR procedure. The meaningful correlation results for the groups were provided in Table 5. In miners, significant moderate negative correlations were most consistently observed between recall performance and network indices in the delta, theta and alpha bands, specifically CC, LE, T and R (e.g., −0.70 ≤ *r* ≤ − 0.48; 0.0009 ≤ *p*_*c*_ ≤ 0.039), indicating that lower network segregation and efficiency in these slow-frequency bands were associated with poorer memory recall. In particular, delta- and theta-band segregation indices (CC, LE, T) showed robust negative associations with recall, suggesting that increased segregation, characterized by stronger within-module but weaker cross-module connectivity, was linked to reduced memory performance. This pattern implies that when functional networks exhibit overly localized connectivity in lower-frequency ranges, inter-regional information transfer, crucial for successful memory retrieval, may be hindered. These results support the notion that optimal cognition depends on a dynamic balance between segregation and integration rather than on the predominance of either. Conversely, in the miner group, higher GE in the beta and gamma bands was positively associated with visuomotor speed and set-shifting performance (TMT-A and TMT-B; 0.51 ≤ r ≤ 0.71; 0.0008 ≤ *p*_*c*_ ≤ 0.029), suggesting that greater high-frequency network integration may facilitate processing speed and cognitive flexibility. We also observed that all indices in the alpha band showed significant associations with psychometric measures. Specifically, GE and T indices were positively correlated with TMT-B and negatively with Recall, respectively, while R, LE, CC, and Q indices also demonstrated meaningful correlations with various cognitive metrics. In controls, significant correlations were found primarily for the R index, which was negatively associated with Recall across alpha, gamma, and full bands (−0.522 ≤ r ≤ −0.496 to, *p*_*c*_ < 0.05). Additionally, in the alpha band, the GE and T indices were negatively correlated with TotalTime and TMT-A, respectively (r = −0.560 and r = −0.530, *p*_*c*_ < 0.05). In groups, a negative association was observed between the alpha-band-specific index T and Stroop performance (Emek et al. 2020), but this effect became non-significant after FDR adjustment. These results suggest that miners exhibit a broader pattern of alpha-band network–cognition associations, while in controls, negative correlations are more restricted and index-specific, highlighting potential compensatory or group-specific neural network mechanisms.

**Table 5:** Statistical correlation results showing meaningful correlations between frequency-band-specific EEG-derived network indices by DTF and ASR at EC state, and psychometric metrics in Miners and Controls (BV: Behavioral Variable, *r*_**:**_ correlation coefficient, *p*_*c*_: corrected p-value with FDR)

	***Miners***				***Controls***			
**f.b**	**Index**	***BV***	***r***	***p***_***c***_	**Index**	***BV***	***r***	***p***_***c***_
δ	CC	Recall	−0,69	0,001				
	LE	Recall	−0,70	0,0009				
	T	Recall	−0,66	0,0023				
	R	Recall	−0,54	0,020				
θ	CC	Recall	−0,60	0,008	Q	ImmediateMemory	0,520	0,026
	LE	Recall	−0,61	0,006				
	T	Recall	−0,57	0,011				
α	GE	TMB	0,63	0,004	GE	TotalTime	−0,560	0,015
	T	Recall	−0,49	0,035	T	TMA	−0,530	0,023
	R	Recall	−0,57	0,013	R	Recall	−0,496	0,036
	LE	Recall	−0,53	0,023				
	CC	Recall	−0,48	0,039				
	Q	TMB	0,59	0,008				
β	GE	TMA	0,51	0,029				
	GE	TMB	0,57	0,012				
γ	GE	TMA	0,63	0,004	R	Recall	−0,485	0,041
	GE	TMB	0,71	0,0008				
	T	TMB	0,51	0,030				
f	Q	TotalTime	0,51	0,028	R	Recall	−0,522	0,026
	Q	TMB	0,46	0,049				
	GE	TMA	0,50	0,032				
	GE	TMB	0,62	0,005				

**Fig. 4:**
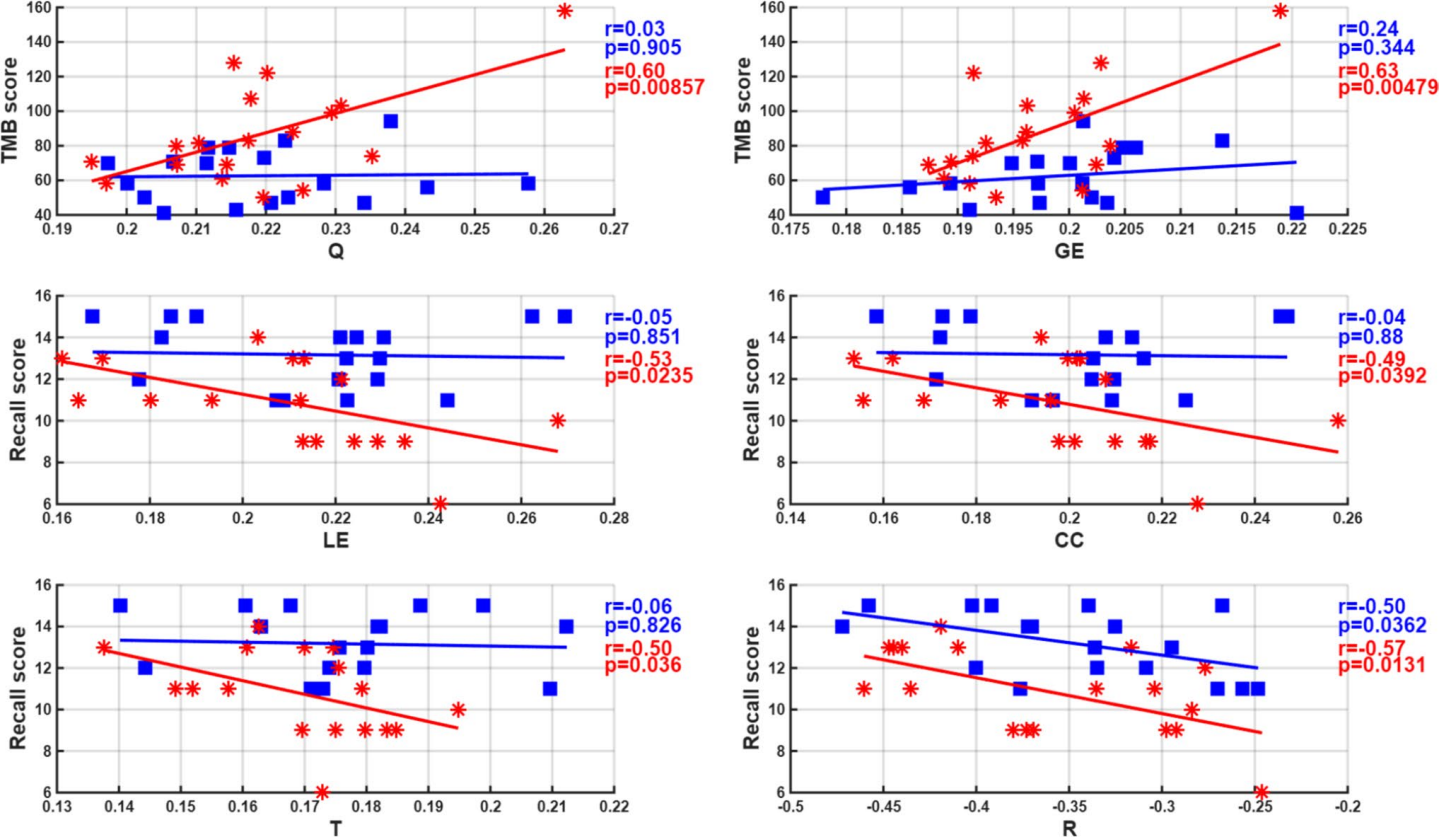
Alpha-band Specific Correlations Between Brain Network Topology and Cognitive Performance in Miners (in red) and Controls (in blue). Scatter plots illustrate the distinct associations between functional connectivity indices and psychometric scores

Since, meaningful correlations were observed between each graph theoretical connectivity index and psychometric scores in alpha band in miners,the divergent relationship between alpha band specific network topologies and cognitive domains was shown in Fig. 4, which highlights the prominent negative correlations between segregation indices (LE, CC, T, R) and memory recall, alongside the positive associations between integration indices (Q and GE) and visuomotor processing speed in the miner group.

## Discussion and conclusion

The primary aim of this study was to quantify potential alterations in brain function among miners who had worked for an average of 10 years under heavy working conditions and shift work, using connectivity indices reflecting brain network segregation, integration, and resilience. These indices are further interpreted in the context of neurodegeneration. To this end, resting-state EEG data in the EO and EC states were analyzed using global brain connectivity methods. Three distinct and widely adopted methods in neuroscience, each with unique strengths, were employed: DTF, which measures directed (causal) and frequency-specific information flow between brain regions by assessing the directionality of neural communication, iCOH, which quantifies neural interactions by excluding spurious connections arising from zero or π phase differences between electrodes; and wPLI, which measures phase synchronization by leveraging the asymmetry of phase differences to minimize spurious connections caused by volume conduction and reference electrode effects.

Technically, the first step of the present study aimed to determine which connectivity approach provides the most reliable estimates in resting-state EEG data and to evaluate the effectiveness of two preprocessing approaches (ICA, ASR) in enhancing data stability. Following a simulation study, EEG series were segmented into 6-s epochs (Miljevic et al. 2025), and then ICCs were computed across repeated measures to assess test–retest reliability within both control and miner groups. Consistent with previous findings highlighting the importance of preprocessing and model selection for EEG connectivity reproducibility (Kessler et al. 2025; Briels et al. 2020), our results showed that the control group exhibited greater overall stability than miners across all methods and frequency bands.

Among the three connectivity approaches, DTF consistently yielded the highest and most reliable ICC values, particularly when combined with ASR preprocessing. In miners, DTF produced excellent ICCs across the full band under ICA, while under ASR, both DTF and iCOH achieved excellent reliability in the high-frequency ranges (beta, gamma, full). Notably, DTF also maintained excellent stability in lower frequencies (delta, theta), underscoring its robustness across frequency bands. In contrast, wPLI generally yielded poor ICCs, consistent with earlier reports suggesting reduced sensitivity to amplitude-related coupling and potential underestimation of directed interactions (Mahjoory et al. 2017). In the control group, the DTF–ASR combination achieved excellent ICCs across all frequency bands, with iCOH also showing excellent stability in high frequencies, reinforcing the superiority of ASR preprocessing for minimizing artifacts and enhancing reproducibility. Further validation of preprocessing efficacy through spectral power comparisons between noisy and cleaned epochs revealed that ASR provided a more effective noise attenuation than ICA, particularly in EC state. Building on this, DTF–ASR was selected as the optimal combination for group-level comparisons. LME modeling revealed significant group differences predominantly in the EC state. Specifically, indices reflecting modularity, integration, and segregation differed between miners and controls in the delta and theta bands, whereas the resilience index showed significant differences across all higher-frequency bands (alpha, beta, gamma, full). The alpha band showed the greatest number of statistically significant indices, and visual inspection confirmed consistent reductions across all indices in miners compared with controls. These findings suggest a broad attenuation of network efficiency and resilience in miners, particularly under resting conditions characterized by internally driven neural dynamics.

The psychometric test results provided in Sect. 2 and the graph-theoretical connectivity analysis summarized in Sect. 3 collectively provide complementary evidence of altered and disorganized cognitive and neurological functioning in miners compared to controls, supporting the hypothesis that occupational stressors in mining impact brain function. Pairwise correlation analysis provides a robust method for assessing statistical relationships between variables for repetitive EEG measurements. Significance was determined using p < 0.05, a standard threshold in neuroscience and psychometric research. The findings that miners are characterized by lower CC compared to controls, and that there is a significant and negative correlation between this index in low frequency bands (delta, theta), representing local connectivity, and verbal memory performance (Recall), are consistent with the literature: In a study estimating graph theoretical CC indices from fMRI data of healthy individuals aged 18–85 (Masuda et al. 2018), and another study computing graph-theoretical connectivity indices from 8-channel surface EEG recordings during resting state and a visual memory task in healthy individuals aged 41–84 (Aydın, 2024), it was commonly shown that, as cognitive performance declines with age, CC indices decrease with advancing age (above 61 years). Similarly, CC indices demonstrate a reduction in the patient group with Alzheimer’s Disease (AD) in analysis of both resting-state EEG data (Aydın, 2024) and visual memory task induced EEG (Lazarou et al. 2022) as well as MRI data (Sharma & Joshi 2025). Likewise, findings of lower CC, particularly in the alpha frequency band, observed in individuals with cognitive impairment (Paitel et al. 2025) are also consistent with the results presented in this study.

The observed concurrent decrease in CC and GE suggests a widespread reduction in both network segregation and integration. From a physiological perspective, decreased CC may reflect weakened local specialization and reduced robustness of clustered processing, whereas decreased GE may indicate diminished long-range communication efficiency and impaired global information transfer. Together, this pattern is consistent with a shift toward a less optimally organized network topology, potentially reflecting reduced functional coordination across distributed neural systems. Importantly, while similar trends of reduced segregation and integration have been reported in neurodegenerative conditions such as Alzheimer’s disease, the present findings should not be interpreted as evidence of shared pathology. Our study was not designed to assess neurodegenerative mechanisms, and conclusions are based solely on graph-theoretical indices derived from resting-state EEG. Therefore, any comparison with Alzheimer’s disease should be viewed as a phenomenological similarity at the level of network metrics rather than as an indication of common etiological processes. Longitudinal and multimodal investigations would be required to determine whether the observed network alterations in coal miners reflect transient functional adaptations, compensatory reorganization, or progressive neural vulnerability.

It is important to note that GE should be interpreted relative to context rather than as a unidirectional marker of cognitive enhancement. Network neuroscience frameworks emphasize that optimal brain function depends on a balanced trade-off between integration and segregation, rather than maximal efficiency per se (Rubinov & Sporns 2010). Accordingly, reduced GE in the present resting-state data likely reflects altered large-scale network organization rather than a simple inverse relationship with cognitive ability.

Correlational analyses further linked other connectivity indices with behavioral and demographic measures. When controlling for age, miners exhibited negative correlations between recall scores and low-frequency (delta and theta) band-specific indices of LE, T, and R, positive correlations between TMT-A and TMT-B scores, and high-frequency (alpha–full) band-specific network efficiency index GE. Compared to controls, the miners exhibited overall lower CC and GE, indicating reduced network segregation and integration capacities. If a brain network has a high average CC value, strong and reciprocal neural communication connections have been established between cortical regions, indicating that local neural circuits are cooperating effectively. High CC values indicate high segregation, an indicator of local information processing efficiency observed in brain regions specialized for specific functions, such as the sensorimotor, visual, or limbic systems. A decrease in CC values, on the other hand, indicates low segregation, suggesting less clustered, more dispersed, or more global connectivity within the cortical network. In such a situation, information flow is thought to be less localized, and therefore, certain functional subnetworks (e.g., attention, motor planning, memory) operate less autonomously. Such a decrease has also been previously reported in neuropsychiatric or neurodegenerative disorders such as dementia, schizophrenia, and post-traumatic conditions. GE, on the other hand, indicates the brain's integration capacity, that is, how efficiently parallel information transmission occurs between different neural populations. GE measures how quickly, over short paths, the network can transfer information between different regions; therefore, decreased GE indicates a slowing of information exchange at the global level and a decrease in the system's integration efficiency. In this study, lower CC and GE values in miners compared to controls indicate that the brain loses efficiency at both the local (segregation) and global (integration) levels. This suggests that chronic workplace stressors (such as hypoxia, shift work, high humidity, and limited space) disrupt both the local and global organization of the network structure. In other words, information processing in miners appears to be neither locally integrated within powerful modules nor effectively integrated across the network. This bidirectional decrease suggests that the brain's capacity for compensatory reorganization is limited, weakening information transfer at both the modular and holistic levels.

Importantly, although miners exhibited reduced GE at the group level compared to controls, variability within the miner group revealed a different pattern. Specifically, individuals with relatively higher GE values showed stronger high-frequency connectivity and poorer executive and processing speed performance. This apparent discrepancy highlights that between-group differences and within-group associations may reflect distinct mechanisms. In this context, relatively higher GE within an overall disrupted network may not indicate optimal integration, but rather a compensatory or maladaptive reorganization characterized by reduced functional specialization and excessive high-frequency coupling. Thus, efficiency should be interpreted relative to the broader network architecture rather than as an inherently beneficial property.

In a retrospective study, it was found that retired individuals from the mining sector had 3.2 times higher healthcare expenses for AD and motor neuron diseases than other individuals living in the same region (Zheng et al. 2020). This suggests that miners are at higher risk of AD at an older age than other occupational groups. In this study, the lower CC observed, especially in the alpha frequency band, may be a potential biomarker for this risk factor observed in miners. Cognitive symptoms such as memory loss and attention deficits, observed in AD and mild cognitive impairment (MCI), can also be present in miners, despite differences in etiology and progression. While AD and MCI are associated with neurodegenerative processes, cognitive impairments in miners typically stem from external factors such as environmental toxins, hypoxia, or traumatic brain injury. Consequently, graph-theoretical analysis results reported in the literature for AD and MCI align with our findings in miners, in which CC values, indicative of brain segregation properties, are significantly altered compared with healthy controls and may serve as precursors to these conditions (Yuan & Zhao 2025). Therefore, early diagnosis of cognitive impairments in miners and preventing exposure to harmful factors can, unlike in AD or MCI, potentially stop or reduce symptom progression.

Detecting early neural system impairments in miners can be valuable not only for understanding the etiology of later-emerging neurodegenerative and motor neuron diseases but also for improving occupational safety by preventing unsafe behaviors. Considering that approximately 90% of occupational accidents are attributed to human factors (Yin et al. 2017), elucidating the neural dynamics underlying fatigue and inattention may play a crucial role in reducing major workplace accidents, particularly when combined with the integration of brain, computer interfaces into occupational settings. Although studies linking miners’ unsafe behaviors with neuroimaging findings are limited, a resting-state fNIRs study employing regional functional connectivity analysis reported that, compared to miners with Non-Unsafe Behavior tendencies, those with Easy-Unsafe Behavior tendencies exhibited lower CC and LE values, particularly in the dorsolateral prefrontal cortex (Tian et al., 2022b). In a high-density (64-channel) EEG analysis using visual stimuli derived from safety-training materials for the coal mining industry (safe vs. hazardous images), theta responses were associated with safer working behaviors (Kuang et al. 2025).

Tables 2 and 3 present the reliability results of connectivity indices estimated across 40 consecutive non-overlapping epochs under EO and EC conditions in terms of ICC values, separately for miners and controls, and processed with either ICA or ASR artifact correction. These analyses evaluate the reliability of repeatedly estimated connectivity metrics. The results show that DTF consistently produced excellent ICC values across all frequency bands and both conditions when combined with ASR, indicating that the ASR method preserves the temporal dynamics and directional relationships critical for DTF-based connectivity estimation in resting-state data (Kaminski & Blinowska 2014; 1991). In contrast, iCOH yielded good-to-excellent ICC values only in controls, particularly with ASR integration. In contrast, it failed to maintain stable estimations in miners at low frequencies (delta and theta bands). This suggests that phase-consistency-based measures such as iCOH are more sensitive to noise and less robust when low-frequency phase synchrony is disrupted (Vinck et al 2011). wPLI, which is designed to reduce volume conduction artifacts by emphasizing non-zero-phase-lag interactions (Vinck et al. 2011), also exhibited limited reliability at lower frequencies in both groups and produced excellent ICCs only in the gamma and full bands in controls. These findings align with previous reports indicating that wPLI’s dependence on consistent phase-lag relationships makes it less stable in low-frequency and noisy EEG data (Vialatte et al. 2007).

When processed with ICA, no excellent ICCs were observed in miners in the low-frequency ranges (delta, theta, alpha). This pattern likely reflects ICA’s tendency to misclassify or overcorrect low-amplitude, physiologically meaningful oscillations as artifacts in resting-state EEG (Onton & Makaig, 2006; Radüntz et al. 2017). ICA is a computational technique used to separate mixed signals into statistically independent sources, often applied to EEG data to remove artifacts (e.g., eye blinks, muscle activity) and isolate neural signals (Hyvärinen & Oja 2000). By decomposing EEG signals into independent components, ICA can enhance the signal-to-noise ratio, potentially improving the detection of true neural connectivity. However, ICA may also alter the phase and amplitude relationships between channels, which can affect connectivity measures differently depending on their mathematical formulations. Automatic ICA component rejection can distort phase and amplitude relationships, thereby reducing the reproducibility of connectivity measures, especially those that rely on phase information, such as iCOH and wPLI (Groppe et al. 2009; Chang et al. 2018). Consequently, the literature indicates that ICA is more commonly used for artifact removal in event-related potential (ERP) studies that rely on trial averaging (Jung et al. 2000; Bell & Sejnowski 1995; Castellanos & Makarov 2006). DTF relies on MVAR model and is sensitive to the temporal dynamics of signals. While ASR preserves these dynamics, ICA’s potential misclassification of certain neural components as noise may reduce DTF’s sensitivity. ICA, which separates signals based on statistical independence, can misidentify information-carrying signals (e.g., low-amplitude gamma-band oscillations) as artifacts when applied to resting-state EEG data, as these oscillations may share statistical properties with artifacts (Onton & Makaig, 2006; Radüntz et al. 2017). Furthermore, ICA may lead to neural information loss in resting-state data due to erroneous component selection, and automatic classification algorithms exhibit higher error rates in unstructured data (Groppe et al. 2009). Another study highlights the risk of ICA misclassifying low-amplitude oscillations as artifacts in resting-state EEG, suggesting that ASR better preserves neural signals in such datasets (Chang et al. 2018; Mullen et al 2015).

DTF is a multivariate, frequency-domain measure based on Granger causality that estimates directed functional connectivity between EEG channels (Kaminski & Blinowska 1991, 2014). DTF relies on temporal dynamics and frequency-domain relationships in the data, while iCOH and wPLI are phase-based connectivity measures designed to minimize volume conduction effects, focusing on non-zero phase-lag interactions (Nolte et al. 2004; Vinck et al. 2011). However, it has been discussed that the performance of wPLI and iCOH methods may be limited in data with low phase consistency (Vinck et al. 2011) and irregular phase relationships (Nolte et al. 2004)**,** respectively. iCOH and wPLI are sensitive to phase synchronization but less dependent on amplitude information, while DTF is sensitive to interrelations in frequency domain. ICA, while removing artifacts, may alter phase relationships by reconstructing signals from independent components, potentially disrupting the synchronization patterns on which iCOH and wPLI rely. Specifically, ICA might attenuate subtle phase-based connectivity differences associated with cognitive impairment, particularly when the impairment manifests in integration states (e.g., global synchronization) rather than in directed interactions. iCOH and wPLI rely on non-zero phase lags and expect moderate phase variability, but miners’ disrupted synchrony may limit their sensitivity. This is consistent with studies showing that connectivity measures vary in their sensitivity to preprocessing steps (Pereda et al. 2005).

Overall, these findings emphasize that ASR provides superior test–retest reliability compared to ICA for estimating resting-state EEG connectivity, particularly for multivariate, directed methods such as DTF. In contrast, phase-based measures (iCOH, wPLI) exhibit lower reliability, especially in conditions of irregular or weak synchronization. This underscores the importance of matching preprocessing strategies to the methodological characteristics of each connectivity approach. While ICA may effectively remove transient artifacts, its aggressive component separation can compromise the temporal and spectral stability required for reliable connectivity estimation.

In conclusion, the DTF–ASR combination emerged as the most robust and reliable approach for assessing resting-state EEG connectivity, offering both high reproducibility and effective noise suppression. The findings highlight distinct neurofunctional patterns between miners and controls, particularly in alpha-band indices at EC state, which may reflect alterations in large-scale brain organization associated with occupational or environmental factors. Collectively, these results underscore the methodological and neurophysiological relevance of directed connectivity metrics and adaptive preprocessing strategies in advancing the reliability of EEG-based network neuroscience.

## Limitations

This study, along with other works (Çelik et al. 2024; 2025), reports that miners show alterations in neurophysiology and impairments in cognitive functioning. These studies were conducted with relatively systematic, controlled, homogeneous, and small sample groups at the recruitment stage. However, underground miners represent a highly heterogeneous population. Within this heterogeneity, positive lifestyle factors such as personality traits, psychological resilience, regular exercise, and healthy nutrition coexist with negative influences such as excessive alcohol and tobacco use or comorbid health conditions, which may further worsen neural outcomes. Therefore, future studies should focus on identifying measures to protect against the neural alterations observed.

In this study, EEG data were recorded using the Enobio32 system (NeuroElectrics, Spain) with the right earlobe serving as the reference electrode. No additional re-referencing was applied. EEG signals are sensitive to the choice of reference; that is, measurements of the same neural activity can vary depending on the reference used. This effect is particularly relevant for sensor-level connectivity analyses. For example, it was emphasized that different reference choices can alter functional connectivity metrics, and therefore, comparisons across studies using different references should be interpreted with caution (Huang et al. 2017). Furthermore, the same authors reported that different re-referencing techniques can affect sensor-level functional connectivity topography (sFCT) and may influence the observed similarities between EEG and MEG data (Huang et al. 2018).

Re-referencing is especially important in clinical and neurological studies, for localizing brain regions, for comparing data across modalities such as EEG and MEG, and for source-level dipole estimation. However, at the sensor level, the impact of re-referencing may be more limited, and in some cases it may not be applied, as in the present study. In sensor-level EEG analyses, the application of re-referencing for graph-theoretical connectivity computations remains a debated topic in the literature, as it depends on the type of reference used and the specific analysis objectives. Therefore, it is crucial to clearly report the reference employed and to consider the potential effects of re-referencing in such analyses.

A methodological consideration concerns the construction of functional networks at the sensor level. Although channel-based graph representations may partially reflect sensor geometry and volume conduction effects, sensor-level network analysis remains a widely adopted and methodologically accepted approach in resting-state EEG research, particularly in studies examining large-scale topological organization and group differences (Klepl et al.^2022^; Jo et al. 2025). Importantly, in the present study, we mitigated zero-lag coupling effects by employing connectivity measures designed to reduce volume conduction sensitivity (iCOH and wPLI) and by incorporating directed modeling (DTF), alongside density-controlled thresholding. Nevertheless, source-reconstructed EEG networks may provide improved spatial specificity and reduce field spread effects (Schoffelen & Gross 2009). Future studies will therefore extend the present framework to anatomically constrained source-space connectivity and graph-theoretical analysis, enabling regionally interpretable network characterization and direct comparison between sensor- and source-level topology within the same cohort.

## Data Availability

The datasets used and/or analysed during the current study are available from the first author on reasonable request.
